# BCL-3 promotes a cancer stem cell phenotype by enhancing β-catenin signalling in colorectal tumour cells

**DOI:** 10.1242/dmm.037697

**Published:** 2019-03-04

**Authors:** Danny N. Legge, Alex P. Shephard, Tracey J. Collard, Alexander Greenhough, Adam C. Chambers, Richard W. Clarkson, Christos Paraskeva, Ann C. Williams

**Affiliations:** 1 Colorectal Tumour Biology Group, School of Cellular and Molecular Medicine, Faculty of Life Sciences, Biomedical Sciences Building, University Walk, University of Bristol, Bristol BS8 1TD, UK; 2 Centre for Research in Biosciences, Faculty of Health and Applied Sciences, University of the West of England, Coldharbour Lane, Bristol BS16 1QY, UK; 3 European Cancer Stem Cell Research Institute, Hadyn Ellis Building, Maindy Road, Cathays, Cardiff CF24 4HQ, UK

**Keywords:** NF-kappaB, NF-κB, LGR5, ASCL2, Wnt, Spheroid, BCL3

## Abstract

To decrease bowel cancer incidence and improve survival, we need to understand the mechanisms that drive tumorigenesis. Recently, B-cell lymphoma 3 (BCL-3; a key regulator of NF-κB signalling) has been recognised as an important oncogenic player in solid tumours. Although reported to be overexpressed in a subset of colorectal cancers (CRCs), the role of BCL-3 expression in colorectal tumorigenesis remains poorly understood. Despite evidence in the literature that BCL-3 may interact with β-catenin, it is perhaps surprising, given the importance of deregulated Wnt/β-catenin/T-cell factor (TCF) signalling in colorectal carcinogenesis, that the functional significance of this interaction is not known. Here, we show for the first time that BCL-3 acts as a co-activator of β-catenin/TCF-mediated transcriptional activity in CRC cell lines and that this interaction is important for Wnt-regulated intestinal stem cell gene expression. We demonstrate that targeting BCL-3 expression (using RNA interference) reduced β-catenin/TCF-dependent transcription and the expression of intestinal stem cell genes *LGR5* and *ASCL2*. In contrast, the expression of canonical Wnt targets Myc and cyclin D1 remained unchanged. Furthermore, we show that BCL-3 increases the functional stem cell phenotype, as shown by colorectal spheroid and tumoursphere formation in 3D culture conditions. We propose that BCL-3 acts as a driver of the stem cell phenotype in CRC cells, potentially promoting tumour cell plasticity and therapeutic resistance. As recent reports highlight the limitations of directly targeting cancer stem cells (CSCs), we believe that identifying and targeting drivers of stem cell plasticity have significant potential as new therapeutic targets.

This article has an associated First Person interview with the first author of the paper.

## Abstract (precis)

**Summary:** BCL-3 acts as a co-activator of β-catenin/TCF-mediated transcriptional activity, driving a stem-cell-like phenotype in colorectal cancer cells, with implications for tumour cell plasticity and therapeutic resistance.

## INTRODUCTION

In 2014, there were around 15,900 deaths attributed to bowel cancer in the UK, placing it second when ranked against all cancer mortalities (Cancer Research UK, 2014). To decrease bowel cancer incidence and improve survival, we need to develop new approaches to cancer treatment. To do this, it is critical that we increase our understanding of the biology of the early stages of human colorectal tumorigenesis. Important recent studies in stem cell biology (Koo and Clevers, 2014) have begun to identify the mechanisms underpinning the drive towards or expansion of mutant stem cells that contribute to the earliest stages of tumour development in mice (Philpott and Winton, 2014). Importantly, environmental factors, including the inflammation and activation of NF-κB signalling, can promote the expansion of mutant cell populations, contributing both towards the earliest stages of tumorigenesis, but also to the maintenance of the tumour, and subsequently to therapeutic resistance and ultimately poor prognosis (Schwitalla et al., 2013; Grivennikov et al., 2010; Vlantis et al., 2011; Shaked et al., 2012).

Unsurprisingly, given its importance in gut development, maintenance and homeostasis (Clevers and Nusse, 2012), deregulation of Wnt signalling is an initiating factor in colorectal tumorigenesis; with adenomatous polyposis coli (*APC*) mutation being the most frequent event in colorectal cancers (CRCs) (Segditsas and Tomlinson, 2006). During active signalling, the Wnt effector protein β-catenin translocates from the cytoplasm to the nucleus and binds T-cell factor (TCF)/lymphoid enhancing-binding factor (LEF) transcription factors situated at promoters of Wnt-responsive genes. β-catenin recruits other co-activators, such as CBP (Li et al., 2007) and BCL-9 (Sustmann et al., 2008), to initiate transcription of genes involved in proliferation or stemness that are otherwise silent in the absence of Wnt ligands (Valenta et al., 2012). Co-activators that bind to the C-terminus of β-catenin are diverse in their methods of transcriptional activation and include chromatin-remodelling enzymes, histone acetyl-transferases and histone methyl-transferases (Valenta et al., 2012). CRCs frequently occur through inactivating mutations of the tumour suppressor adenomatous polyposis coli (APC) protein (part of the ‘destruction complex’ that degrades β-catenin) or, less frequently, via stabilising mutations in β-catenin itself (Cancer Genome Atlas Network, 2012), consequently resulting in deregulated β-catenin signalling. Although there are many proteins that interact with β-catenin to influence its role in the cell (Valenta et al., 2012), it was of interest that a solitary panel in a figure of a paper by Kim et al. suggested that β-catenin may interact with the NF-κB co-regulator B-cell lymphoma 3 (BCL-3) (either directly or indirectly), although the significance of the interaction was not described (Kim et al., 2005).

The BCL-3 protein is highly expressed in a subset of CRCs, in which we have recently shown that it inhibits apoptosis and promotes tumour growth (Urban et al., 2015). The *BCL3* gene was first discovered through cloning and sequencing of recurring t(14;19)(q32.3;q13.1) translocations identified in chronic lymphocytic leukaemia patients (McKeithan et al., 1990). It was predicted to encode a protein with a molecular weight of around 47 kDa, with a proline-rich N-terminal domain, seven central tandem-repeat cdc10 domains (ankyrin repeat domains), and a serine- and proline-rich C-terminal domain (Ohno et al., 1990). BCL-3 is an atypical member of the inhibitor of kappa B (IκB) family of proteins and has been demonstrated to modulate transcription of NF-κB target genes via binding to homo-dimeric subunits of p50 or p52 through its ankyrin repeat domains (Wulczyn et al., 1992; Bours et al., 1993). The p50/p52 subunits possess DNA-binding motifs, known as the Rel homology domain, enabling them to occupy κB sites at promoters of NF-κB-responsive genes (Pereira and Oakley, 2008). This permits BCL-3 to activate (through its own transactivation domain or via recruiting alternative co-activators) or repress gene transcription (Dechend et al., 1999).

Under homeostatic conditions, BCL-3 plays important roles in the immune system and regulation of inflammation. Evidence of these functions were provided by *Bcl-3*-knockout (*Bcl-3*^−/−^) mice, which display defects in germinal centre development, a failure to generate IFN-γ-producing T cells and an inability to produce antigen-specific antibodies in response to infection by certain bacterial species (Schwarz et al., 1997; Franzoso et al., 1997). Interestingly, *Bcl-3*^−/−^ mice treated with dextran-sodium sulphate (DSS) develop less severe colitis compared to wild-type (WT) mice (O'Carroll et al., 2013); however, it has also been demonstrated that BCL-3 suppressed expression of pro-inflammatory cytokines in macrophages, dendritic cells and B cells in response to lipopolysaccharide (LPS)-mediated Toll-like receptor (TLR) activation (Carmody et al., 2007), thereby demonstrating a complex role for BCL-3 in the regulation of inflammation.

Although first characterised in hematopoietic cancers (Ohno et al., 1990), there is an emerging role for BCL-3 in solid tumours. It has been implicated in cancers arising in multiple tissue types, including breast (Cogswell et al., 2000), prostate (Ahlqvist et al., 2013), cervical (Maldonado et al., 2010) and colorectal (Urban et al., 2015). BCL-3 bears numerous tumour-promoting capabilities, such as increasing proliferation (Na et al., 1999) and inflammation (Chang and Vancurova, 2014), inhibiting apoptosis (Kashatus et al., 2006), and promoting metastasis (Wakefield et al., 2013). Importantly, studies by Puvvada et al. and more recently by Saamarthy et al. report that around 30% of colorectal tumours present with nuclear BCL-3 (Puvvada et al., 2010; Saamarthy et al., 2015).

Considering the importance of Wnt/β-catenin and NF-κB crosstalk in cellular de-differentiation and tumour initiation in the intestine (Schwitalla et al., 2013), surprisingly there have been no studies exploring the role of the NF-κB co-regulator BCL-3 in Wnt signalling in CRC. Here, we report that BCL-3 is an important co-activator of β-catenin/TCF-mediated transcriptional activity in CRC cells. We show that BCL-3 regulates β-catenin-mediated transcription and expression of colorectal stem cell and cancer stem cell (CSC) marker genes *LGR5* and *ASCL2*, functionally promoting a stem cell phenotype in CRC cells.

We propose that BCL-3 acts as a driver of the stem cell phenotype in CRC cells, potentially promoting tumour cell plasticity and therapeutic resistance. As recent reports highlight the limitations of directly targeting CSCs, we believe that identifying and targeting drivers of stem cell plasticity (Shimokawa et al., 2017) for their use as new therapeutic targets has significant potential.

## RESULTS

### β-catenin regulates BCL-3 expression in CRC cells

**Fig. 1.**
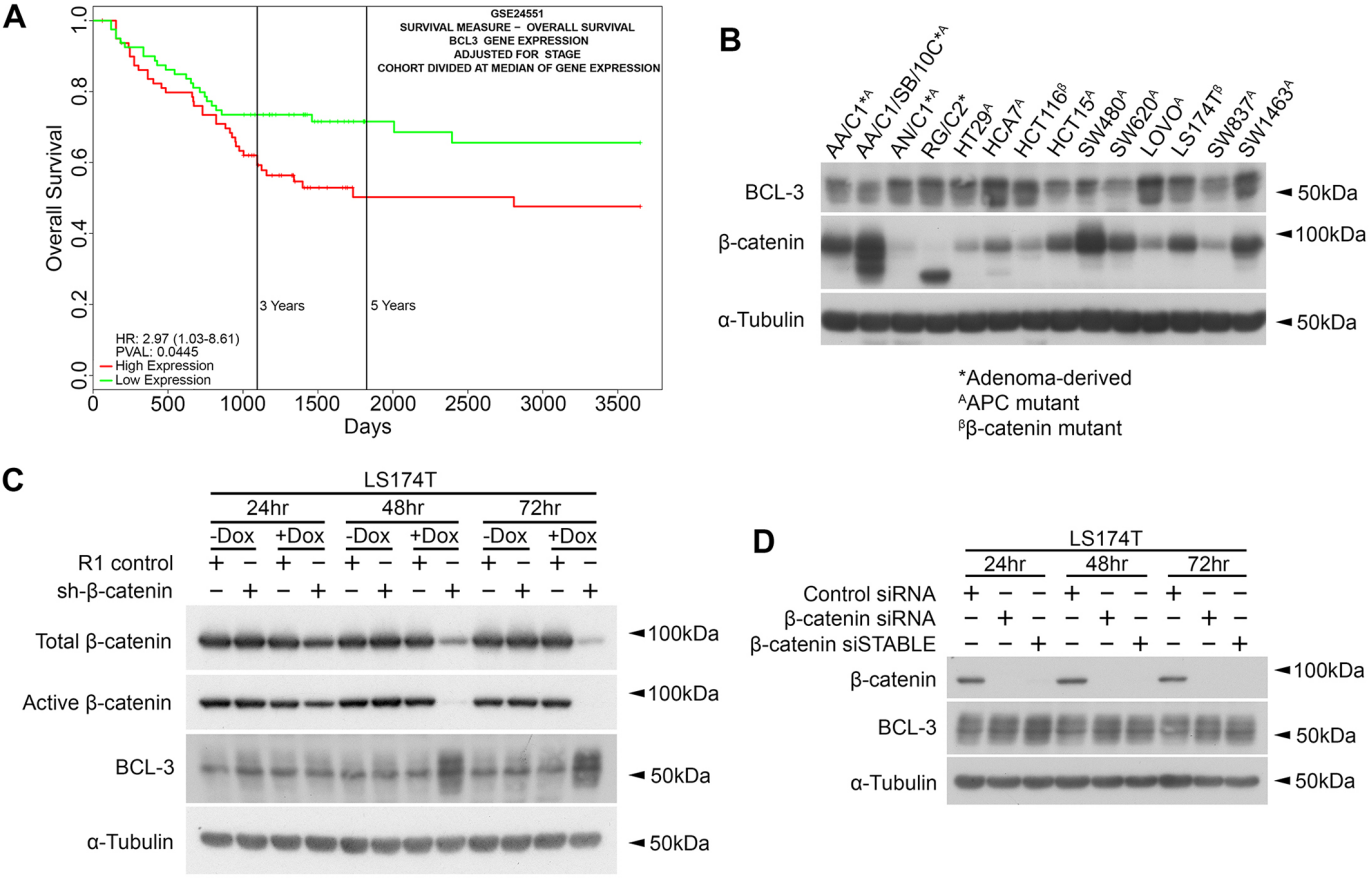
**β-catenin regulates *BCL-3* expression in CRC cells.** (A) Survival analysis in relation to *BCL-3* expression generated using a publicly available CRC dataset (GSE24551) and Progene V2 (Goswami and Nakshatri, 2014). (B) Western blot analysis of adenoma- and carcinoma-derived colorectal cell lines showing expression of BCL-3 and β-catenin. α-tubulin serves as a loading control. (C) Western analysis of total and active β-catenin and BCL-3 expression in LS174T cells with dox-inducible expression of β-catenin shRNA following 24, 48 and 72 h of dox treatment (1 µg/ml). LS174T/R1 cells possess a dox-responsive promoter upstream of a scrambled shRNA sequence and express a non-targeted shRNA upon treatment with dox. α-tubulin serves as a loading control. (D) Western analysis of β-catenin and BCL-3 expression in LS174T cells at 24, 48 and 72 h post-β-catenin siRNA transfection (25 nM). β-catenin siSTABLE is a β-catenin-targeted siRNA with enhanced stability. α-tubulin serves as loading control. Dox, doxycycline.

To initially demonstrate the importance of BCL-3 expression for patient outcome in CRC, we carried out survival analysis in relation to BCL-3 expression by using a publicly available CRC dataset (GSE24551; https://www.ncbi.nlm.nih.gov/geo/query/acc.cgi?acc=GSE24551) and Progene V2 (Goswami and Nakshatri, 2014); results are displayed in Fig. 1A. Survival analysis in this dataset revealed that high BCL-3 expression was linked to significantly reduced survival when adjusted for tumour stage. To examine the role of BCL-3 in Wnt/β-catenin signalling, we first screened a panel of human adenoma- and carcinoma-derived cell lines for expression of BCL-3 and β-catenin (Fig. 1B). Results show that both adenoma- and carcinoma-derived cell lines express β-catenin and BCL-3 protein, although there is an apparent inverse correlation between β-catenin and BCL-3 protein levels (those cells with lowest β-catenin generally having higher levels of BCL-3). To determine whether BCL-3 is regulated by β-catenin expression, LS174T cells with a doxycycline-inducible shRNA targeted towards β-catenin (sh-β-catenin; a kind gift from Professor Hans Clevers, Hubrecht Institute, Utrecht, The Netherlands) were used to suppress β-catenin expression. LS174T/R1 control cells were transfected with an otherwise identical plasmid, which expressed a non-targeting shRNA upon doxycycline addition. Protein levels of β-catenin and BCL-3 were analysed by western blot following 72 h of doxycycline treatment. Results are displayed in Fig. 1C. Doxycycline addition in the LS174T/sh-β-catenin cells resulted in efficient suppression of total and active β-catenin from 48 h, with expression even further reduced by 72 h. No reduction in β-catenin protein was detected in sh-β-catenin cells without doxycycline treatment. Interestingly, BCL-3 expression was strongly induced following β-catenin suppression at the 48 and 72 h time points following doxycycline addition, suggesting that BCL-3 expression is repressed by Wnt/β-catenin signalling.

As off-target effects are possible when using siRNA or shRNA to target mRNAs (Jackson and Linsley, 2010), LS174T cells were selected and transfected with two independent siRNA sequences targeting β-catenin. One of these siRNAs (β-catenin siSTABLE) has enhanced stability within the cell. Cells were treated with control and β-catenin siRNA for 72 h. Expression of BCL-3 was analysed by western blot (Fig. 1D). Efficient β-catenin suppression was observed from 24 h onwards with both β-catenin-targeting siRNAs. BCL-3 upregulation was detected in response to β-catenin suppression with both sequences and at all time points analysed, in agreement with results in Fig. 1C. Together, these results show that BCL-3 expression is increased following β-catenin suppression.

### BCL-3 interacts with β-catenin and regulates β-catenin/TCF reporter activity in CRC cell lines

**Fig. 2.**
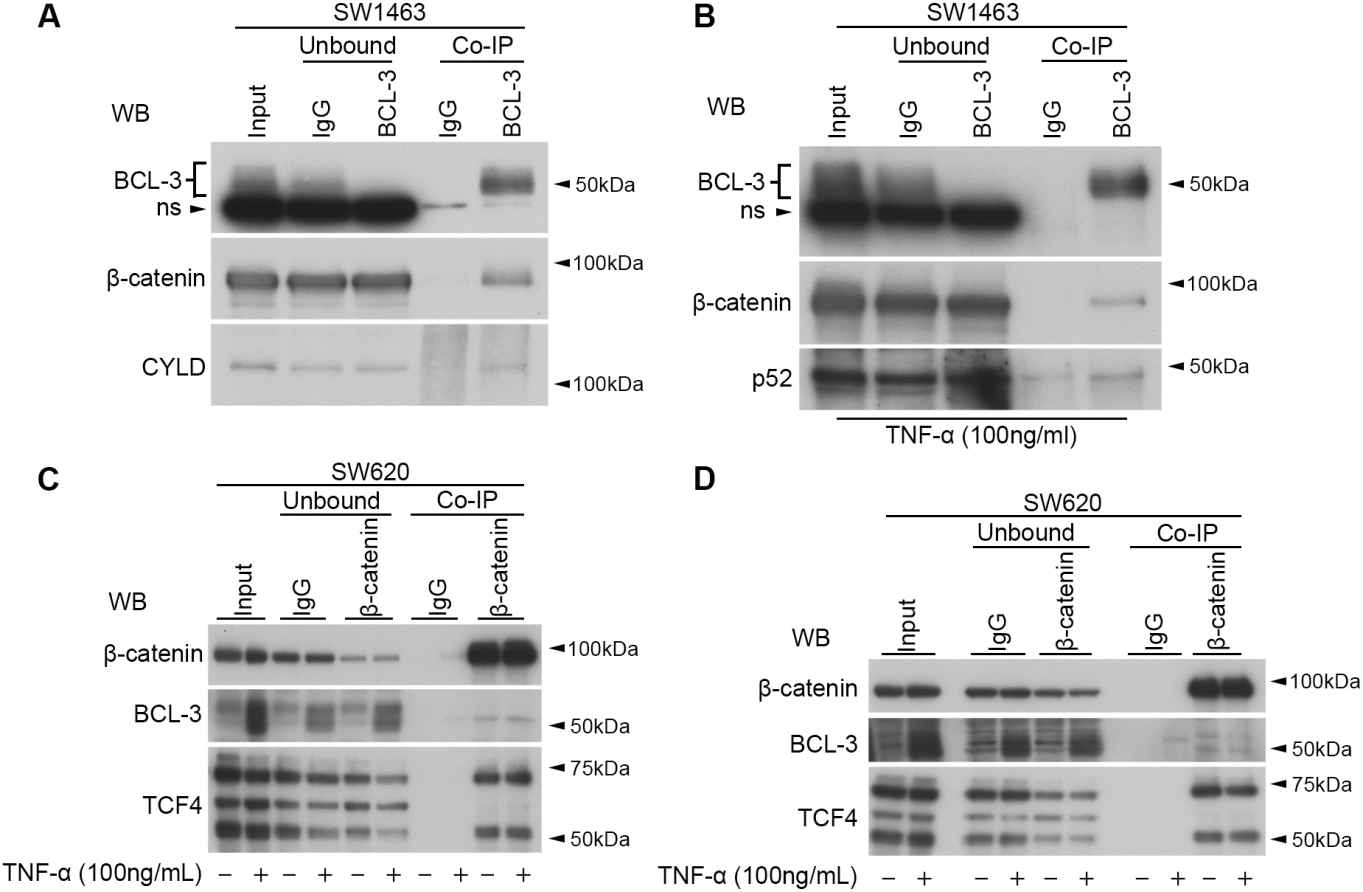
**BCL-3 interacts with β-catenin in CRC cell lines.** (A,B) BCL-3 Co-IP performed in SW1463 nuclear-enriched lysates. Unbound (immuno-depleted) lysates show depletion of proteins after IP. Immunoprecipitates were analysed by western blot for BCL-3 and β-catenin. IgG serves as a negative control. (A) CYLD serves as a positive control for BCL-3 binding. (B) Cells were treated with 100 ng/ml TNF-α for 6 h prior to lysis. Immunoprecipitates were additionally analysed for p52. Note the presence of a non-specific (ns) band in BCL-3 western analysis with use of Abcam anti-BCL-3 antibody. (C,D) Nuclear β-catenin Co-IPs in SW620 cells. Panels C and D represent experimental replicates. Western analysis of non-treated and 6-h TNF-α-treated cells following β-catenin Co-IP. TCF4 serves as a positive control for β-catenin binding. Mouse pan-IgG serves as a negative control. Unbound, immuno-depleted lysates following Co-IP; WB, western blot antibody.

To investigate any potential interaction between BCL-3 and β-catenin in CRC cells, we selected the *APC*-mutant SW1463 cell line for its relatively high endogenous expression of both BCL-3 and β-catenin. We performed BCL-3 protein complex immunoprecipitations (Co-IPs) on nuclear-enriched lysates and were able to identify an interaction between endogenous BCL-3 and β-catenin that had not been previously reported in CRC cells (Fig. 2A). Ubiquitin carboxyl-terminal hydrolase CYLD (CYLD) was included as a positive control of BCL-3 binding. In addition, as it has previously been demonstrated that TNF-α can induce BCL-3 binding to p52 homodimers (Zhang et al., 2007), we treated SW1463 cells with TNF-α for 6 h to activate NF-κB signalling and carried out BCL-3 Co-IPs in the resulting lysates. In stimulated cells, we detected an interaction between BCL-3 and p52 and again demonstrated the association of BCL-3 and β-catenin (Fig. 2B). The interaction was further detected by β-catenin Co-IPs in control and TNF-α-treated SW620 cells (Fig. 2C,D; TCF4 was included as a positive control for β-catenin interaction). These data suggest that endogenous BCL-3 interacts with β-catenin in CRC cells.

**Fig. 3.**
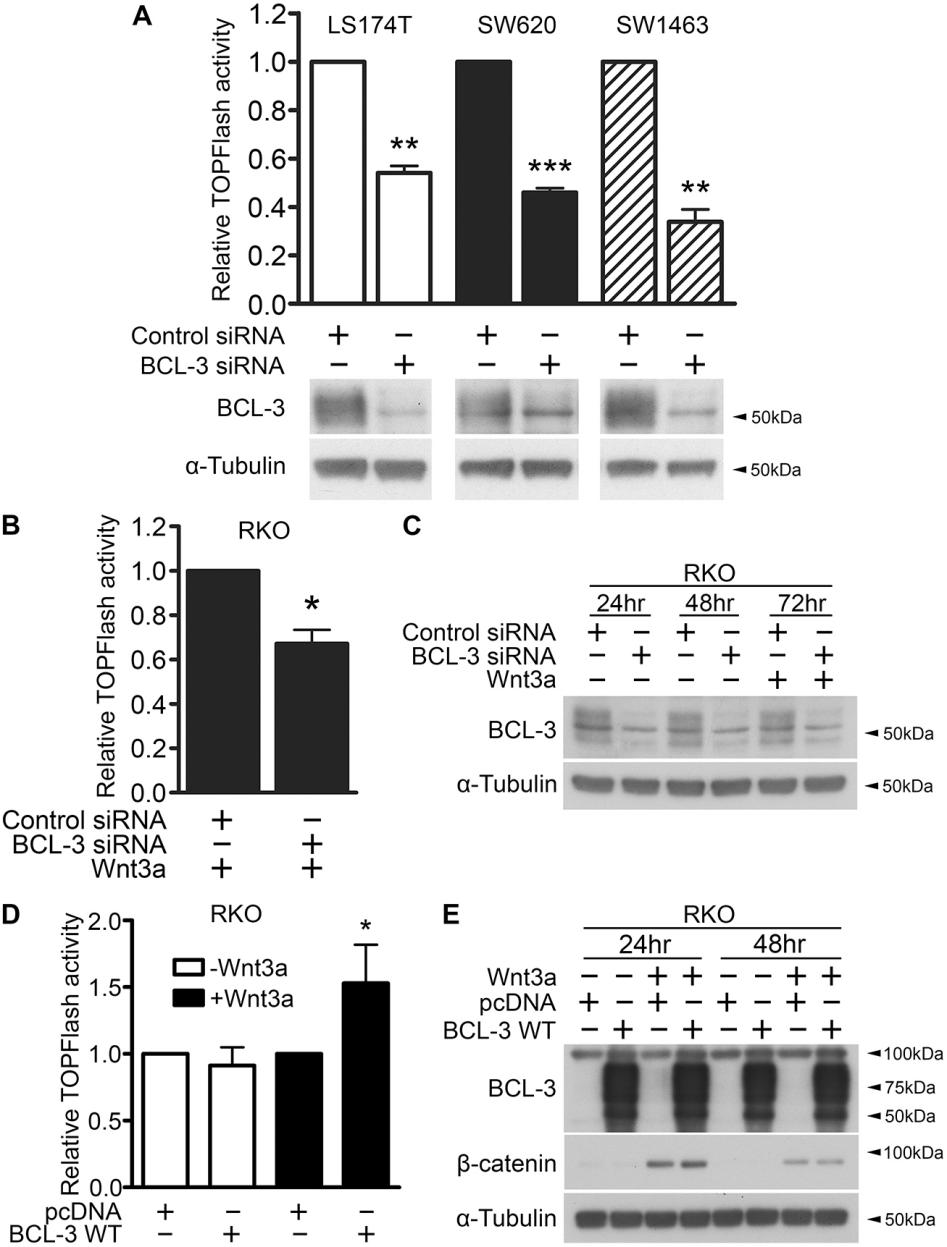
**BCL-3 regulates β-catenin/TCF reporter activity.** (A) β-catenin/TCF (TOPFlash) luciferase reporter assay. TOPFlash reporter activity was measured 72 h post-siRNA transfection in LS174T, SW620 and SW1463 carcinoma cells. Results are expressed as TOPFlash/Renilla. *N*=3, ±s.e.m. **P<0.01, ***P<0.001. Western analysis shows expression of BCL-3. α-tubulin serves as a loading control. (B) TOPFlash reporter activity measured 72 h post-siRNA transfection in RKO cells. 100 ng/ml Wnt3a was added to cells at 48 h post-siRNA transfection. Results are expressed as TOPFlash/FOPFlash. One-sample *t*-test. *N*=3, ±s.e.m. *P<0.05. (C) Western analysis of BCL-3 expression over the duration of the TOPFlash assay performed in B. α-tubulin serves as a loading control. (D) TOPFlash reporter activity measured 48 h following BCL-3 overexpression and Wnt3a (100 ng/ml) treatment. Results are expressed as TOPFlash/FOPFlash. One-sample *t*-test. *N*=4, ±s.d. *P<0.05. (E) Western analysis of BCL-3 and β-catenin expression over the duration of the TOPFlash assay performed in D. α-tubulin serves as a loading control.

We next investigated the effects of BCL-3 expression on β-catenin/TCF-mediated transcription. To do this, we used siRNA to suppress *BCL3* expression in colorectal cell lines before transfecting cells with TOPFlash reporter plasmid to measure β-catenin/TCF-mediated transcriptional output. Interestingly, we discovered a significant decrease in TOPFlash activity in LS174T (colon-derived, mutant β-catenin), SW620 (lymph-node-derived, mutant APC) and SW1463 (rectal-derived, mutant APC) cell lines (Fig. 3A). These data indicate that BCL-3 can regulate β-catenin/TCF-mediated transcription in CRCs with common Wnt driver mutations. In addition, we examined the role of BCL-3 in RKO CRC cells, which are reported to harbour no activating Wnt pathway mutations and show no detectable TOPFlash activity under unstimulated conditions (da Costa et al., 1999). In agreement with preceding experiments, there was a significant decrease in Wnt3a-induced TOPFlash activity in RKO cells when BCL-3 expression was suppressed (Fig. 3B,C). We next analysed the outcome of transient BCL-3 overexpression in CRC cells. Overexpression of BCL-3 in SW620 and LS174T cell lines harbouring activating Wnt pathway mutations did not show any regulation of TOPFlash reporter activity (data not shown). The same was true in unstimulated RKO cells. However, in RKO cells stimulated with Wnt3a, BCL-3 overexpression significantly enhanced β-catenin/TCF reporter activity (Fig. 3D,E). These findings show that, in a non-deregulated Wnt setting, BCL-3 can modulate β-catenin/TCF-dependent transcription, suggesting that Wnt3a-mediated transcriptional responses are enhanced by BCL-3.

### BCL-3 regulates expression of stemness-associated Wnt targets

**Fig. 4.**
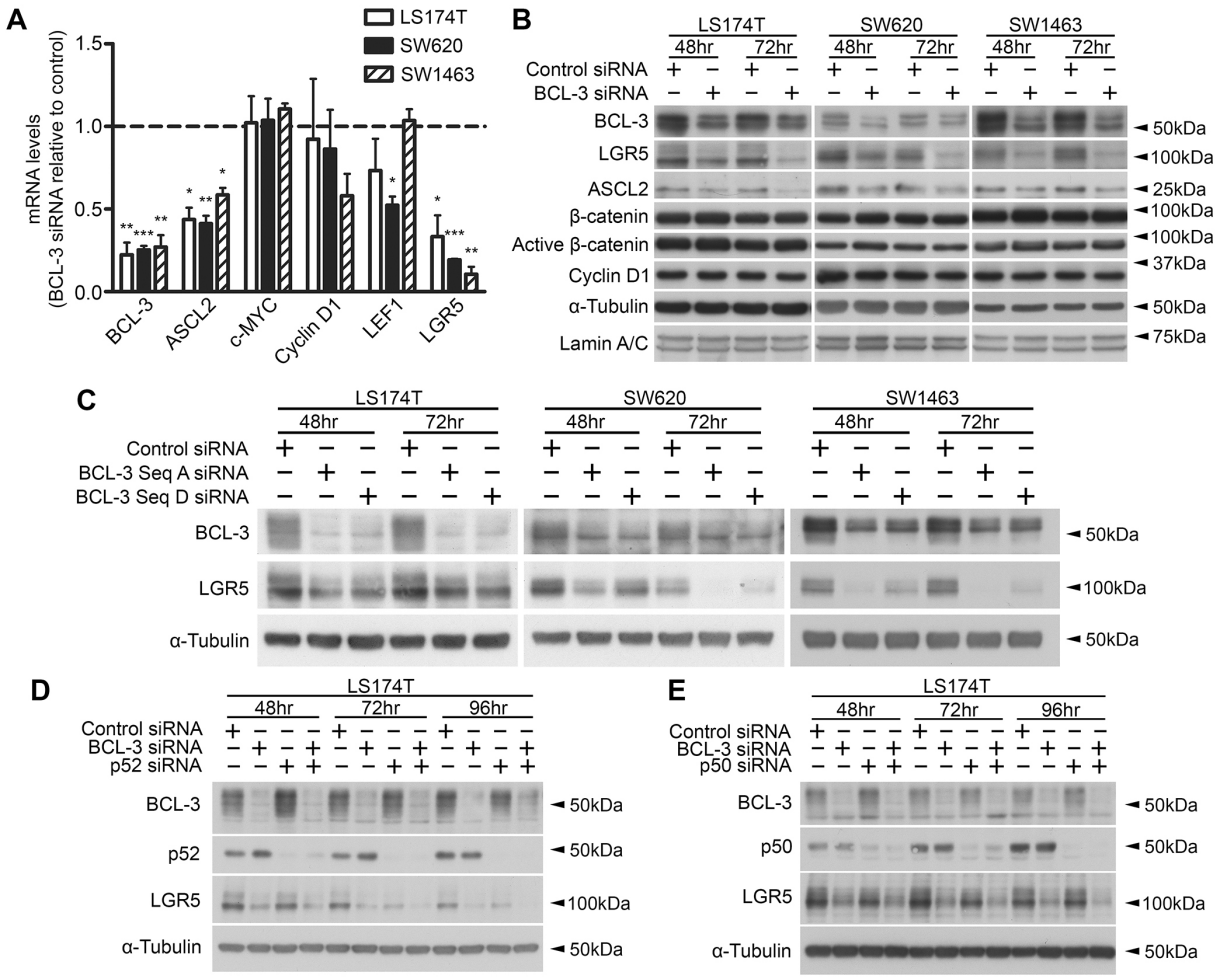
**BCL-3 regulates expression of stemness-associated Wnt targets.** (A) Quantitative reverse transcriptase-PCR (qRT-PCR) mRNA analysis of Wnt target gene expression in LS174T, SW620 and SW1463 cells following BCL-3 suppression. Data shows mRNA expression of genes normalised to housekeeping genes at 72 h post-siRNA transfection, showing *BCL-3*-knockdown cells relative to controls. One-sample *t*-test. *N*=3, ±s.e.m. *P<0.05, **P<0.01, ***P<0.001. (B) Western analysis of LGR5 and ASCL2 expression in LS174T, SW620 and SW1463 cells following BCL-3 suppression. Expressions of total β-catenin, de-phosphorylated (active) β-catenin and cyclin D1 are also shown. α-tubulin serves as a loading control. ASCL2 expression is shown in nuclear-enriched lysates, with lamin A/C serving as a loading control. (C) LGR5 expression in LS174T, SW620 and SW1463 cells following BCL-3 suppression with two independent siRNA sequences. LGR5 and BCL-3 expression was analysed by western blot. α-tubulin serves as a loading control. (D) Western analysis of BCL-3, p52 and LGR5 expression in LS174T cells at 48, 72 and 96 h following siRNA transfection with the indicated siRNAs (50 nM). α-tubulin serves as a loading control. (E) Western analysis of LS174T cells for expression of BCL-3, p52 and LGR5 at 48, 72 and 96 h following siRNA transfection with the indicated siRNAs (50 nM). α-tubulin serves as a loading control.

After establishing that BCL-3 regulates β-catenin/TCF-dependent transcription, we investigated the role of BCL-3 in Wnt target gene regulation. Wnt targets are heavily involved in the maintenance of stem cells in the colon (Clevers and Nusse, 2012), with *LGR5* and *ASCL2* providing key examples of genes that are expressed in intestinal stem cells, but not expressed in other cell types of the gut (Sato et al., 2009; van der Flier et al., 2009; Barker et al., 2007; Schuijers et al., 2015). With this in mind, we used quantitative reverse transcriptase PCR (qRT-PCR) to analyse mRNA levels of some classical Wnt target genes – along with stemness-associated Wnt targets – on BCL-3 suppression in LS174T, SW620 and SW1463 cells. We found that classical β-catenin/TCF targets such as MYC and cyclin D1 were not significantly regulated by BCL-3 knockdown, whereas the stem-cell-specific Wnt target genes *LGR5* and *ASCL2* were significantly downregulated in all three cell lines (Fig. 4A). Western analysis was used to measure LGR5 and ASCL2 protein expression at 48 and 72 h post-*BCL-3* siRNA transfection; we found clear downregulation of LGR5 and ASCL2 protein in all cell lines and at both time points (Fig. 4B). Interestingly, we saw no decrease in total or ‘actively signalling’ (de-phosphorylated) β-catenin levels. *LGR5* encodes a G protein-coupled receptor expressed in stem cells of the intestine and is also thought to identify CSCs in CRC (Barker et al., 2007; Merlos-Suárez et al., 2011; Kemper et al., 2012; Hirsch et al., 2014). To further confirm regulation of LGR5 by BCL-3 – and to rule out any potential off-target effects of using a single siRNA sequence – we used a second *BCL-3*-targeting siRNA. LGR5 suppression by BCL-3 depletion was demonstrated using two independent siRNA sequences in the SW620, LS174T and SW1463 cell lines (Fig. 4C), indicating that BCL-3 regulates LGR5 expression in CRC cells with different mutational backgrounds. This result confirmed that BCL-3-mediated intestinal stem cell marker regulation was not the product of siRNA-mediated off-target effects. Using LS174T and SW620 cell lines stably expressing a BCL-3 expression construct (termed LS174T^BCL-3­^ and SW620^BCL-3^) or empty vector (LS174T^pcDNA^ and SW620^pcDNA^), we were able to show that enhanced expression of BCL-3 moderately increased LGR5 expression (Fig. S1). Together, these data suggest that BCL-3 may be promoting β-catenin/TCF-dependent transcription at specific intestinal stemness-associated gene loci, which does not appear to be via increasing the pool of actively signalling β-catenin.

To investigate the role of NF-κB homodimers in BCL-3-mediated regulation of LGR5, we used RNAi to co-suppress BCL-3 and p50 – and subsequently BCL-3 and p52 – in LS174T cells (Fig. 4D,E). BCL-3 was still able to regulate LGR5 expression in both p50- and p52-depleted cells. These results indicate that BCL-3-mediated regulation of LGR5 is independent of NF-κB p50/p52 homodimers.

### BCL-3 does not mediate β-catenin activity through promoting nuclear translocation or altering levels of LEF1

**Fig. 5.**
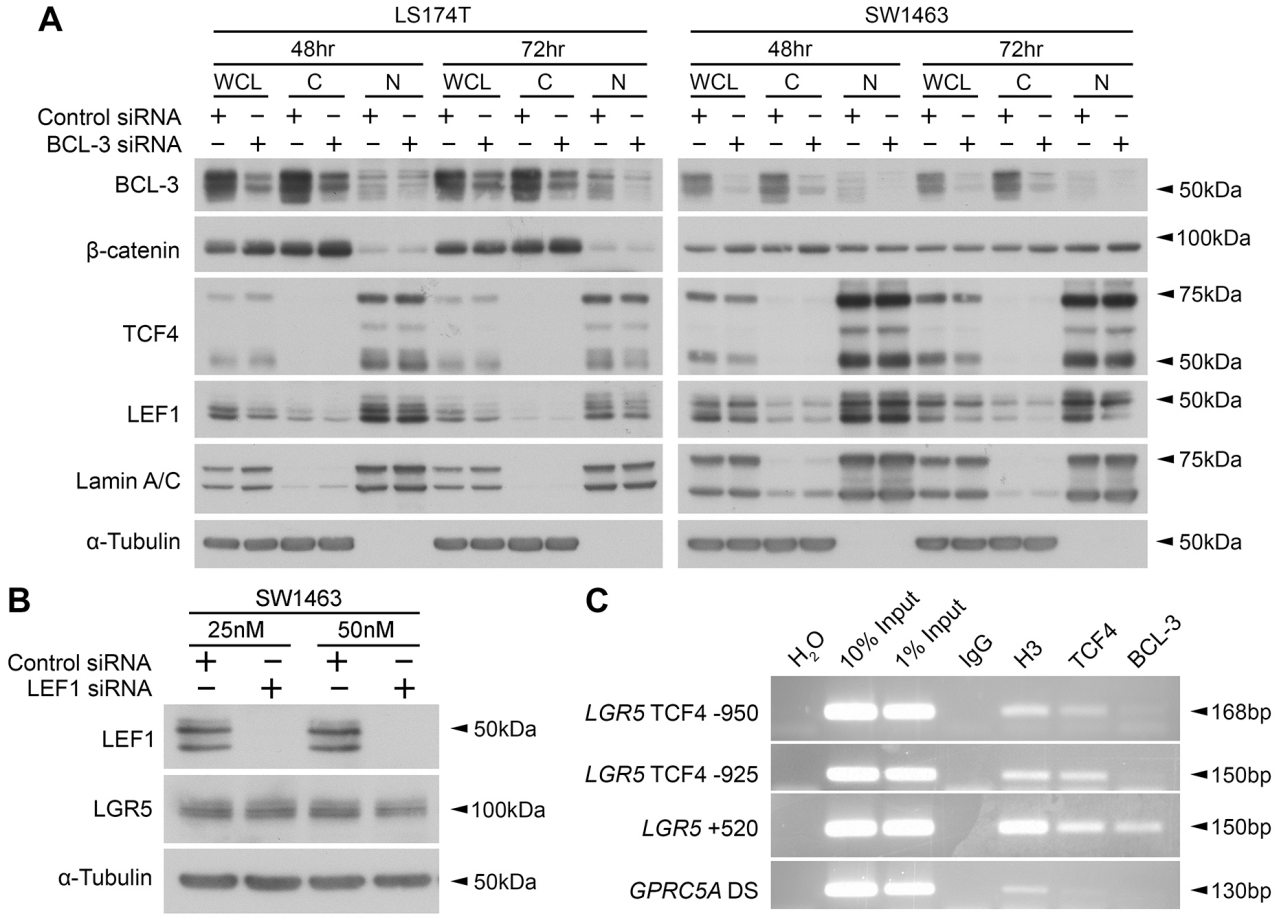
**BCL-3 does not mediate β-catenin activity through promoting nuclear translocation or altering levels of LEF1.** (A) Nuclear/cytoplasmic enrichments of LS174T and SW1463 cells following BCL-3 suppression, showing localisation and expression of the Wnt transcriptional regulators β-catenin, TCF4 and LEF1. Western analysis confirms BCL-3 suppression. α-tubulin and lamin A/C serve as loading controls and confirm cytoplasmic/nuclear enrichment. WCL, whole cell lysate; C, cytoplasm-enriched lysate; N, nuclear-enriched lysate. (B) LGR5 expression following LEF1 suppression with 25 nM and 50 nM siRNA in SW1463 cells. Data shows 48 h post-siRNA transfection. α-tubulin serves as a loading control. (C) ChIP analysis of three sites in the *LGR5* promoter in SW1463 cells for presence of TCF4 and BCL-3. H_2_O serves as a negative control for PCR. IgG serves as a negative control for immunoprecipitation (IP). Acetylated histone H3 serves as a positive control for IP. *GPRC5A* DS primers were designed to a downstream region in the *GPRC5A* gene with no reported transcription-factor binding and serves as a negative primer control.

Having shown nuclear interaction between BCL-3 and β-catenin, and given that BCL-3 has been shown to promote NF-κB nuclear translocation (Zhang et al., 1994), to further examine how BCL-3 regulates β-catenin/TCF activity and target gene expression we investigated whether BCL-3 enhances β-catenin nuclear localisation. We performed western analysis on LS174T and SW1463 nuclear/cytoplasmic-enriched lysates following BCL-3 suppression. BCL-3 knockdown did not affect β-catenin nuclear translocation in either cell line (Fig. 5A). We also analysed expression levels of TCF4 and LEF1, as diminished nuclear levels of these transcription factors may have a profound effect on transcription of Wnt target genes. We found no consistent regulation of TCF4, but a small decrease in LEF1 expression (Fig. 5A). *LEF1* is a Wnt target gene and encodes a transcription factor that mediates β-catenin signalling (Hovanes et al., 2001; Filali et al., 2002). To investigate the role of LEF1 in BCL-3-mediated LGR5 regulation, we used RNAi to suppress LEF1 in SW1463 cells and analysed LGR5 expression using western blotting. We found that LEF1 knockdown had no effect on LGR5 levels (Fig. 5B). These findings signify that modulation of β-catenin/TCF-dependent transcription and regulation of stemness-associated Wnt targets by BCL-3 is not through promotion of β-catenin, TCF4 or LEF1 nuclear translocation. When taken alongside our data from Co-IP and TOPFlash experiments (Figs 2, 3), these results support the idea that BCL-3 may be acting as a transcriptional co-activator of the β-catenin/TCF complex. To test this, we performed BCL-3 and TCF4 chromatin immunoprecipitation (ChIP) in SW1463 cells using primers designed to specific regions in the *LGR5* promoter. *LGR5* was first demonstrated as a β-catenin/TCF4 target gene by Van der Flier et al. (2007); primers were designed to encompass TCF4 consensus sites (Hoverter and Waterman, 2008) in the *LGR5* promoter region. The *LGR5* TCF4−950 and the *LGR5* TCF4−925 primers span a TCF4-binding site around 950 bp upstream of the *LGR5* transcriptional start site (TSS). The *LGR5* +520 primer set span a region of 150 bp from around 500 bp in a distal region downstream of the *LGR5* TSS, a potential regulatory region for LGR5 transcription, as determined by using TCF4 ChIP-seq and H3K4Me1 methylation data tracks on the UCSC Genome Browser (http://genome.ucsc.edu/) (Kent et al., 2002) as a reference. The *GPRC5A* downstream (DS) primer set is designed to a 150 bp region located 30,760 bp downstream of the *GPRC5A* TSS. This primer set was used as a negative control as it was designed to a non-promoter/non-enhancer region of a non-relevant gene (Greenhough et al., 2018).

Immunoprecipitation was performed using antibodies to BCL-3, IgG (negative control) and positive controls, acetylated histone H3 and TCF4. Acetylated histone H3 is a marker of permissive, or ‘active’, chromatin, from which genes can be transcribed (Workman and Kingston, 1998; Yan and Boyd, 2006). Results are displayed in Fig. 5C. TCF4 was located at all three regions of the *LGR5* promoter analysed and was absent from the *GPRC5A* downstream region. BCL-3, however, was not detected at the −950 bp or −925 bp regions. Interestingly, BCL-3 could be detected at the +520 bp region, suggesting an increase in β-catenin/TCF4 activity via binding to this region in CRC cells. The high levels of histone H3 acetylation at the +520 bp region are consistent with those seen at enhancer regions in other sites of the genome (Creyghton et al., 2010). Together, these results suggest that BCL-3 can be detected at the +520 bp region of the *LGR5* promoter, consistent with its role as a transcriptional co-activator.

### BCL-3 regulates colorectal spheroid and tumoursphere formation in 3D culture

**Fig. 6.**
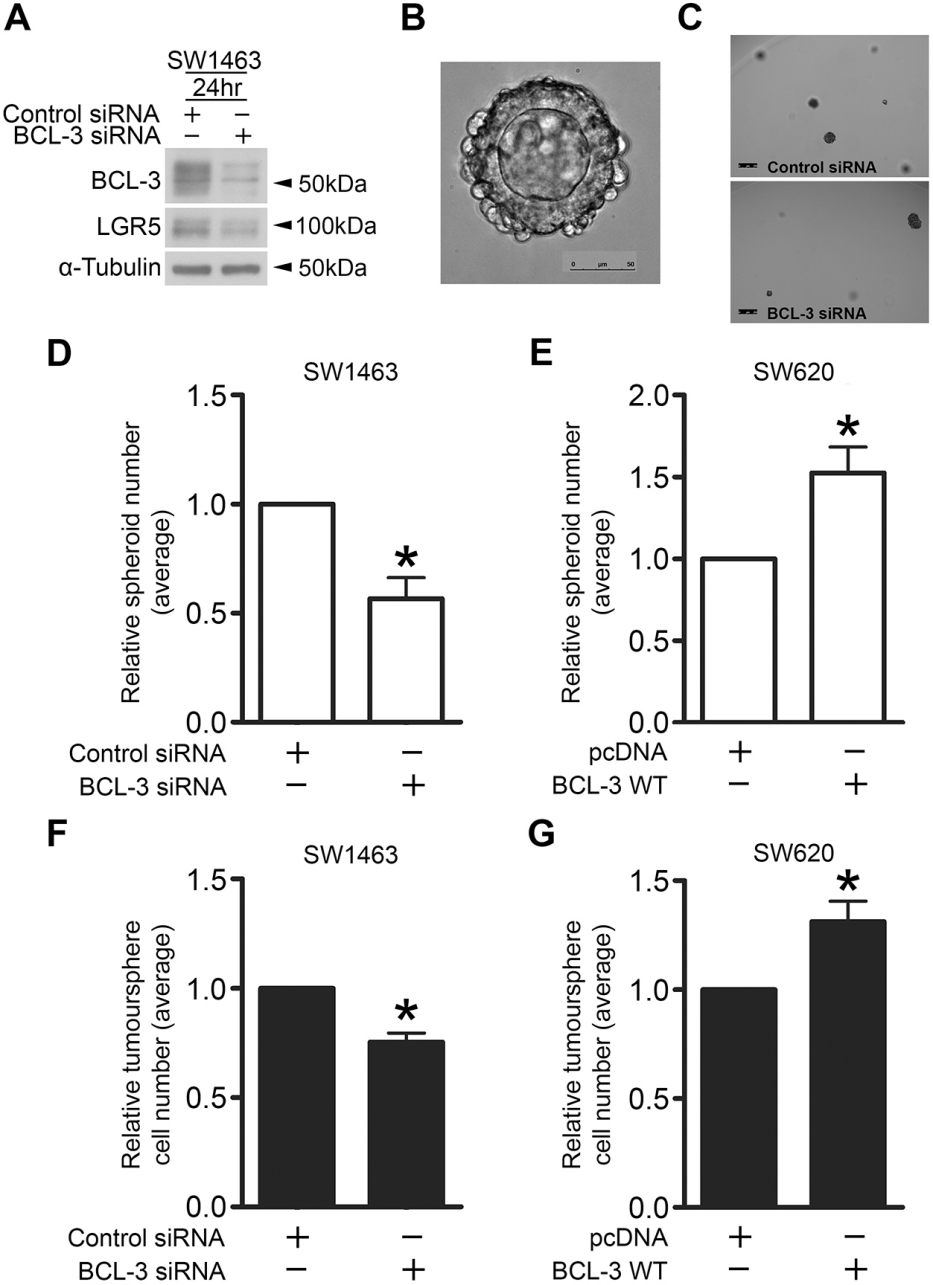
**BCL-3 regulates colorectal spheroid and tumoursphere formation.** (A) Western analysis confirming BCL-3 suppression and LGR5 downregulation in SW1463 cells seeded into Matrigel. α-tubulin serves as a loading control. (B) Widefield microscopy image of a colorectal spheroid grown from a single SW1463 cell following 10 days of culture. 20× objective. Scale bar: 50 µm. (C) Widefield microscopy images of wells containing *BCL-3* knockdown and control SW1463 spheroids. 5× objective. Scale bars: 250 µm. (D,E) Spheroid-formation assay. (D) SW1463 cells transfected with *BCL-3* siRNA or negative control siRNA were re-suspended in Matrigel and seeded into 48-well plates at 24 h post-transfection. Spheroids were cultured for 10 days as previously described (Sato et al., 2011). Wells were imaged and analysed using Matlab R2015a software, with subjective gating applied to exclude any cells/debris less than 3000 µm^2^ in cross-sectional area. Control siRNA, 9.37±4.51 spheroids; *BCL-3* siRNA, 5.78±4.02 spheroids. One-sample *t*-test. *N*=3, ±s.e.m. *P<0.05. (E) SW620 cells stably transfected with pcDNA-BCL-3 WT vector (SW620^BCL-3^) or empty pcDNA vector (SW620^pcDNA^) as a negative control were seeded into Matrigel, cultured and analysed as in D. pcDNA, 4.15±2.48 spheroids; BCL-3 WT, 5.88±2.40 spheroids. One-sample *t*-test. *N*=4, ±s.e.m. *P<0.05. (F,G) Tumoursphere-formation assay. (F) SW1463 cells transfected with *BCL-3* siRNA or negative control siRNA were re-suspended in tumoursphere medium and cultured as described previously (Ricci-Vitiani et al., 2007). Tumourspheres were dissociated and cells counted following 6 days of culture. Control siRNA, 8.93×10^4^±3.95×10^4^ tumourspheres; *BCL-3* siRNA, 6.61×10^4^±2.63×10^4^ tumourspheres. One-sample *t*-test. *N*=3, ±s.e.m. *P<0.05. (G) SW620 cells stably transfected with pcDNA-BCL-3 WT vector or empty pcDNA vector as a negative control were seeded, cultured and analysed as in F. pcDNA, 3.83×10^4^±2.38×10^4^ tumourspheres; BCL-3 WT, 4.81×10^4^±2.48×10^4^ tumourspheres. One-sample *t*-test. *N*=4, ±s.e.m. *P<0.05.

Given that BCL-3 promotes LGR5 and ASCL2 expression, we investigated the functional consequence of stemness marker regulation using an adapted version of the organoid model system pioneered by Sato et al. (2009) to grow CRC cell lines as 3D spheroids. It has been shown that single LGR5-positive cells can form organoids in Matrigel (Sato et al., 2009; Barker et al., 2010). As BCL-3 promotes LGR5 expression, we hypothesised that suppressing BCL-3 may inhibit the ability of single cells to progress and form spheroids. SW1463 cells were used as they form luminal spheroids in 3D culture (Fig. 6B), suggesting the presence of differentiated cell types in addition to CSCs (Yeung et al., 2010; Ashley et al., 2013). Additionally, SW1463 cells express high levels of BCL-3 and undergo efficient BCL-3 suppression following siRNA transfection, increasing the likelihood of observing a strong phenotypic effect. Equal numbers of viable control- and *BCL-3*-siRNA-treated cells were re-suspended in Matrigel and seeded into 48-well plates. We then measured the number of spheroids formed after 10 days of culture. At the time of seeding, duplicate flasks were lysed and checked for efficacy of *BCL-3* knockdown. We found that suppression of BCL-3 and downregulation of LGR5 was achieved after 24 h (Fig. 6A), and strong suppression of BCL-3 was maintained following 84 h of *BCL-3* knockdown (Fig. S2), ensuring that BCL-3 was suppressed during the critical early stages for spheroid initiation. Furthermore, we noted a significant reduction in the number of spheroids formed upon BCL-3 suppression relative to controls following 10 days of growth (Fig. 6B-D). We repeated this experiment using SW620^pcDNA^ and SW620^BCL-3^ cells to investigate the outcome of BCL-3 overexpression on spheroid formation. SW620 cells were chosen for BCL-3 overexpression experiments in 3D conditions as they have low endogenous levels of BCL-3; we hypothesised that overexpressing BCL-3 in a low endogenously expressing cell line would have a greater effect than overexpressing BCL-3 in SW1463 cells, which naturally express high endogenous levels of BCL-3 (enough endogenous protein to form functional interactions). Of note, these cells were maintained on selection medium to ensure high constitutive overexpression of BCL-3 for the duration of the experiment (Fig. S1). We found that BCL-3 overexpression significantly increased the number of spheroids formed following 10 days of culture (Fig. 6E). These results suggest that BCL-3 enhances the ability of cells to initiate full spheroid formation under these conditions, indicating that BCL-3 may promote stemness of CRC cells.

To further investigate the effect of BCL-3 on stemness of CRC cells, we used a tumoursphere 3D culture system. Tumoursphere assays have been used previously to identify undifferentiated CSCs in CRC (Ricci-Vitiani et al., 2007). We transfected SW1463 cells with control or *BCL-3* siRNA for 24 h before seeding into serum-free conditions in non-adherent 6-well plates and cultured for 6 days. The number of viable cells were counted at the end of this period to determine any effect that BCL-3 might have on tumoursphere formation. Consistent with the findings above, we found that BCL-3 suppression significantly reduced tumoursphere formation in serum-free conditions (Fig. 6F). Furthermore, upon BCL-3 overexpression in the same system using SW620^pcDNA^ and SW620^BCL-3^ lines, we reported a significant increase in the number of cells making up the tumourspheres formed by *BCL-3*-overexpressing cells versus controls (Fig. 6G). This suggests that, by enhancing their ability to form tumourspheres in 3D culture conditions, BCL-3 enhances the stem-like potential of CRC cells.

## DISCUSSION

Given that BCL-3 is overexpressed in a subset of CRCs and is linked to poor prognosis (Puvvada et al., 2010), and that the Wnt pathway is deregulated in the vast majority of colorectal tumours (Segditsas and Tomlinson, 2006), the aim of this study was to investigate the role of the NF-κB co-regulator BCL-3 in β-catenin/TCF-mediated signalling in CRC cells. Previously, survival of *APC* mutant stem cells had been shown to be promoted by NF-kB signalling (van der Heijden et al., 2016). However, initial Co-IP experiments demonstrated an interaction between BCL-3 and β-catenin, with further work illustrating BCL-3-mediated regulation of TOPFlash reporter activity and the intestinal stem cell genes *LGR5* and *ASCL2*. Furthermore, this regulation was found to be independent of NF-κB homodimers, highlighting a novel link between NF-κB co-regulator BCL-3 and β-catenin/TCF-mediated signalling. Finally, colorectal spheroid and tumoursphere formation assays indicated that BCL-3 plays a functional role in enhancing stem-like potential of CRC cells.

Importantly, we show that BCL-3 suppression can downregulate β-catenin signalling in cells expressing mutant *APC* (the most common mutation in CRCs) and mutant β-catenin itself (in addition to cells with non-mutated Wnt components), which suggests that targeting BCL-3 would reduce β-catenin signalling in colorectal tumours. The ‘just right’ model for Wnt signalling in CRC was suggested by Albuquerque et al., who observed the non-random distribution of mutational hits in *APC* in tumours from familial adenomatous polyposis (FAP) patients (Albuquerque et al., 2002). It was shown that some β-catenin-binding activity (and resulting β-catenin degradation) in one of the *APC* alleles was always retained, suggesting an optimal level of β-catenin/TCF-mediated transcription for tumour progression – as mutations that completely abolished the β-catenin-binding ability of *APC* were not selected for (Albuquerque et al., 2002). By suppressing BCL-3, it is tempting to speculate that this might reduce the Wnt signalling level to below the ‘just right’ threshold in colorectal tumour cells, preventing deregulated transcription of select Wnt target genes.

In this report, we demonstrate that BCL-3 regulated the expression of the β-catenin/TCF targets *LGR5*, *ASCL2* and, to a lesser extent, *LEF1*. Conversely, expression of other canonical β-catenin/TCF targets analysed (cyclin D1 and MYC) was not modulated by BCL-3. The BCL-3-mediated regulation of a specific subset of intestinal stem cell and Wnt target genes, and not of proliferation-inducing Wnt targets, is unusual given that suppression of BCL-3 expression inhibits total β-catenin activity, as shown by TOPFlash reporter activity in CRC cells. However, transcriptional regulation of Wnt target genes from genomic DNA is more complex than the simple binding of TCF/LEF to DNA and availability of free β-catenin to activate transcription. The vast number of proteins that interact with β-catenin to regulate its transcriptional activity – with some co-regulators only regulating a subset of Wnt targets – supports this (Valenta et al., 2012). Preferential regulation of stem-cell-specific targets is not unheard of and has been shown recently in colorectal tumours with *PTPRK-RSPO3* fusions. Tumours harbouring gene fusions involving *RSPO2* and *RSPO3* were found to occur in around 10% of colorectal tumours and result in upregulation of *RSPO2* or *RSPO3* expression. Additionally, these tumours do not contain other Wnt-activating mutations (Seshagiri et al., 2012). Upon treatment of these tumours with anti-RSPO3 antibody using patient-derived xenograft models, expression of *LGR5* and *ASCL2* were highly downregulated, whereas *MYC*, *AXIN2* and *CCND1* (cyclin D1) did not rank among the top 100 downregulated genes (Storm et al., 2016). Additionally, previous work has shown that the Wnt co-activator BCL9/9L promotes activation of stemness-related target genes and not of proliferation-inducing Wnt targets (Moor et al., 2015). As yet, the mechanism behind the selectivity of *LGR5* and *ASCL2* regulation over other canonical Wnt targets remains undefined and warrants further investigation. It may be governed by other co-regulators present in the transcriptional complex, as has been previously demonstrated by Kim et al., who showed that both activation and repression of *KAI1* occurred in the presence of BCL-3, with activation or repression being dependent on the co-regulator Pontin or Reptin, respectively (Kim et al., 2005).

By preferentially diverting β-catenin/TCF-mediated transcription towards the stemness genes *LGR5* and *ASCL2* over some of the more classical Wnt targets such as cyclin D1 and MYC, it appears that BCL-3 may be driving CRC cells towards a stem-cell phenotype. Not only are *LGR5* and *ASCL2* robust stem cell markers, but numerous studies have shown them to be upregulated in CRCs, with many reporting that they are markers for CRC stem cells (Merlos-Suárez et al., 2011; Kemper et al., 2012; Kobayashi et al., 2012; Hirsch et al., 2014; Jubb et al., 2006; Ziskin et al., 2013; Shimokawa et al., 2017). The CSC hypothesis states that there are cells within tumours that are capable of self-renewal, in addition to producing other heterogeneous, differentiated cell types that constitute the tumour mass (Clarke et al., 2006). CSCs fuel tumour growth and are thought to be responsible for tumour re-constitution in cases of relapse if they are not eradicated by initial treatments (Merlos-Suárez et al., 2011; Beck and Blanpain, 2013). Therefore, CSCs must be eliminated by future therapeutics for CRC. In this study, enhancing levels of BCL-3 in CRC cells promoted colorectal spheroid and tumoursphere formation, indicating an increase in CRC stem-like activity (Ricci-Vitiani et al., 2007). Similarly, BCL-3 suppression impaired the ability of single cells to progress to fully formed spheroids and inhibited tumoursphere formation. Through using colorectal spheroid- and tumoursphere-formation assays, we present functional evidence that BCL-3 is promoting CSC activity. Intriguingly, our findings complement those of a recent study by Chen et al. proposing that BCL-3 plays a key role in maintaining naïve pluripotency in mouse embryonic stem cells (Chen et al., 2015). The findings of the Chen et al. study, albeit in a non-cancer background, concur with our conclusion that BCL-3 is important in promoting stem-like activity. Crucially, factors promoting plasticity of CSCs will be important for future therapeutics, as reversion of differentiated progeny to CSCs expressing LGR5 in CRC has been identified as a barrier to treatment (Shimokawa et al., 2017).

Recent work has provided insight into limitations on the efficacy of directly targeting LGR5^+^ CRC stem cells in primary tumours. Using genetic reporters and lineage tracing, Shimokawa et al. (2017) revealed that consecutive, specific ablation of LGR5^+^ CSCs in xenotransplanted CRC organoids led to the reversion of differentiated KRT20^+^ cells. These cells filled the niche vacated by the LGR5^+^ cells and began to re-express LGR5, causing initial decreases in tumour volume but eventual tumour reconstitution following cessation of treatment (Shimokawa et al., 2017). Data from this study suggest that BCL-3 may play a role in the de-differentiation and reconstitution of the tumour. Targeting BCL-3 may be an effective mechanism to prevent the reversion of LGR5^+^ cells in colorectal tumours. This could be a way of targeting cellular plasticity and a means of maintaining inhibition of primary tumour growth in already established primary tumours, without the associated tissue toxicity caused by sustained direct targeting of LGR5 (Tian et al., 2011). Furthermore, there is evidence that targeting cellular plasticity can be effective – a study targeting BMI-1-expressing cells showed that this reduced the number of cancer-initiating cells *in vivo* (Kreso et al., 2014). In addition, BMI-1 cells have been shown to produce LGR5^+^ cells following epithelial damage (Tian et al., 2011). Targeting niche factors that promote stemness and plasticity, such as BMI-1 and BCL-3, may be an effective way to target CRC stem cells in primary tumours and prevent reversion of differentiated cells to LGR5^+^ CSCs.

In terms of therapeutics, BCL-3 could be an exciting novel target for CRC therapy. The *Bcl-3*^−/−^ mouse develops normally and has no obvious intestinal phenotype (Schwarz et al., 1997), alluding to potentially fewer toxic side effects than targeting the β-catenin/TCF interaction directly, which is crucial for normal gut homeostasis and crypt maintenance (Valenta et al., 2012; Clevers and Nusse, 2012). Our data show that BCL-3-mediated regulation of LGR5 and ASCL2 is especially important in CRC. Furthermore, we would hypothesise that modulation of these targets by BCL-3 could play a role in other physiological circumstances. Stresses such as inflammation may increase BCL-3 expression (Brasier et al., 2001; Tohyama et al., 2017), resulting in enhanced Wnt signalling; with the link between inflammation and Wnt signalling having been previously established (Schwitalla et al., 2013). Current studies are focusing on revealing any potential implications for normal intestinal physiology through inflammation-induced regulation of BCL-3 expression. Data from this study in RKO cells (Fig. 3) with a WT Wnt pathway suggest that targeting this protein may help to prevent or reduce aberrant Wnt signalling at early stages of tumorigenesis, in addition to later-stage cancers. Furthermore, BCL-3 appears to still modulate β-catenin/TCF-mediated transcription downstream of mutated APC (SW1463, SW620) and β-catenin (LS174T), two of the most frequent mutations in CRC (Cancer Genome Atlas Network, 2012). If the BCL-3/β-catenin interaction could be mapped and disrupted, this may be an effective therapeutic approach for targeting CSC plasticity in CRC and may add to the current arsenal of Wnt-related inhibitors currently under development (Katoh, 2017).

In conclusion, we have shown for the first time that BCL-3 potentiates β-catenin/TCF-mediated signalling in CRC, and selectively regulates transcription of intestinal stem cell genes and Wnt targets *LGR5* and *ASCL2*, promoting a CSC phenotype. Our data suggest that BCL-3 may represent an exciting new avenue for targeting plasticity of CSCs in CRC, particularly as it enhances β-catenin activity downstream of mutations in APC and β-catenin that occur frequently in CRC.

## MATERIALS AND METHODS

### Cell lines

All cell lines used in this study were obtained from American Type Culture Collection (ATCC; Rockville, MD, USA). LS174T cells were established from a Duke's type B colonic adenocarcinoma; SW620 cells were derived from a lymph node metastasis of the primary colorectal tumour (Duke's type C colorectal adenocarcinoma); SW1463 cells were established from a Duke's type C rectal adenocarcinoma; RKO cells were established from a colorectal carcinoma. All cell lines were routinely tested for mycoplasma contamination using MycoAlert PLUS mycoplasma detection kit (Lonza, MD, USA).

### Cell culture

All cell lines were cultured in Dulbecco's modified Eagle medium (DMEM) (Gibco, Life Technologies, Paisley, UK) with added 10% foetal bovine serum (FBS) (GE Healthcare, UK), 2 mM glutamine (Sigma-Aldrich, Dorset, UK), 100 units/ml penicillin and 100 units/ml streptomycin (Invitrogen, Life Technologies, Paisley, UK). LS174T^pcDNA/BCL-3^ and SW620^pcDNA/BCL-3^ cell lines were cultured in 10% DMEM with added 400 µg/ml G418 (Sigma-Aldrich). For stock purposes, cells were maintained in T25 flasks (Corning, NY, USA) and incubated at 37°C in dry incubators maintained at 5% CO_2_. Cells media were changed every 3-4 days.

### SDS-PAGE and western analysis

Cell lysates were prepared and subjected to western analysis as described previously (Williams et al., 1993) using antibodies to the following: α-tubulin (T9026; Sigma-Aldrich; 1:10,000), ASCL2 (4418; EMD Millipore, Watford, UK; 1:500), β-catenin (9587; Cell Signaling Technology, MA, USA; 1:5000), β-catenin (610153; BD Biosciences, CA, USA; 1:10,000), de-phosphorylated (actively signalling) β-catenin (05-665; EMD Millipore; 1:1000), BCL-3 (ab49470; Abcam, Cambridge, UK; 1:1000), BCL-3 (23959; Proteintech, Manchester, UK; 1:2000), MYC (sc-40; Santa Cruz Biotechnology, TX, USA; 1:500), cyclin D1 (2978; Cell Signaling Technology; 1:1000), lamin A/C (4200236; Sigma; 1:5000), LEF1 (2230; Cell Signaling Technology; 1:1000), LGR5 (ab75850; Abcam; 1:1000), NF-κB1 (p50; sc-8414; Santa Cruz Biotechnology; 1:1000), NF-κB2 (p52;05-361; EMD Millipore; 1:1000) and TCF4 (2569; Cell Signalling Technology; 1:1000).

### Complex immunoprecipitation (Co-IP)

BCL-3 CoIPs were performed in SW1463 nuclear-enriched lysates as previously described (Petherick et al., 2013). Briefly, 500 µg of rabbit pan-IgG (12-370; EMD Millipore)-pre-cleared nuclear-enriched protein lysates were cleared with 6 µg of anti-BCL-3 antibody (23959; Proteintech) conjugated to Dynabeads Protein A beads (Invitrogen) before undergoing western blot analysis. Beads were retrieved using a Dyna-Mag 2 magnet (Invitrogen). Immunoprecipitates were analysed by western blot for BCL-3 (ab49470; Abcam) and β-catenin (610153; BD Biosciences). For TNF-α-treated CoIPs, 100 ng/ml TNF-α (Source BioScience, Nottingham, UK) was added to cells for 6 h prior to lysis. Immunoprecipitates were additionally analysed for p52 (05-361; EMD Millipore).

### Gene knockdown via RNA interference (RNAi)

Cell lines were reverse transfected in Opti-MEM (Gibco) using RNAiMax (Invitrogen) with 50 nM of siRNA, unless otherwise stated. Individual sequences and relevant non-targeting controls were used for silencing *BCL3*, whereas a smart pool and non-targeting controls were used to silence *LEF1*, *NFKB1* and *NFKB2*. All siRNA sequences were produced by Dharmacon (GE Lifesciences).

### Quantitative reverse transcriptase-PCR (qRT-PCR)

Tri-Reagent (Sigma-Aldrich) was added to cells and an RNAeasy mini kit (Qiagen, Limberg, Netherlands) was used according to the manufacturer's instructions to clean up RNA before synthesis of cDNA and qRT-PCR were performed as previously described (Petherick et al., 2013) using the following primers: *BCL3* (cat. no. QT00040040), *CTNNB1* (β-catenin) (cat. no. QT01331274), *CMYC* (cat. no. QT00035406), *CCND1* (cyclin D1) (cat. no. QT00495285), *LEF1* (cat. no. QT00021133) and *LGR5* (cat. no.QT00027720) (all from Qiagen). Gene expression was normalised to housekeeping gene *TBP* (cat. no. QT00000721; Qiagen). *ASCL2* primer sequences (Sigma) were obtained from Jubb et al. (2006) (forward 5′-GGCACTGCTGCTCTGCTA-3′, reverse 5′-GTTCACGCTCCCTTGAAGA-3′).

### Generation of stable BCL-3-expressing cell lines

Cells were transfected in Opti-MEM (Gibco) using Lipofectamine 2000 (Invitrogen) with pcDNA3-BCL-3 WT vector (Keutgens et al., 2010; Viatour et al., 2004) (kindly provided by Alain Chariot, University of Liège, Belgium; re-cloned by Tracey Collard, University of Bristol, UK) or empty pcDNA3 vector as a control. Resistant clones were selected and pooled in media supplemented with 500 µg/ml neomycin.

### TCF reporter assay (TOPFlash reporter assay)

BCL-3 expression was suppressed via RNAi and TOPFlash assay was performed as previously described (Petherick et al., 2013) using Promega Dual-Luciferase Reporter Assay System (Promega, WI, USA) according to the manufacturer's instructions. Luminescence was measured at 560 nm using a Modulus luminometer (Turner Biosciences, CA, USA). Co-transfection of FOPFlash reporter with mutated TCF consensus sites was used alongside TOPFlash to monitor non-specific output. In RKO cells, 100 ng/ml recombinant human WNT3a protein (R&D Systems, Abingdon, UK) was added to cells 48 h post-siRNA transfection. For BCL-3 transient overexpression, cells were transfected with pcDNA3 control and pcDNA3-BCL-3 WT plasmids prior to Wnt3a treatment.

### Spheroid-formation assay

For *BCL-3*-knockdown experiments, equal numbers of single SW1463 cells transfected with *BCL-3* siRNA or negative-control siRNA were re-suspended in Matrigel (Becton Dickinson, Oxford, UK) and seeded into 48-well plates (Corning) at 24-h post-transfection. Matrigel was submerged in spheroid medium consisting of Advanced DMEM:F12 (Gibco), 0.1% BSA (Sigma-Aldrich), 2 mM glutamine (Sigma-Aldrich), 10 mM HEPES (Sigma-Aldrich), 100 units/ml penicillin, 100 units/ml streptomycin, 1% N2 (Gibco), 2% B27 (Gibco) and 0.2% N-acetyl-cysteine (Gibco). Spheroids were cultured for 10 days as previously described (Sato et al., 2011). Wells were imaged using a DMI6000 widefield microscope (Leica Microsystems, Wetzlar, Germany) and Leica LAS-X acquisition software (Leica Microsystems). Images were analysed using Matlab R2015a software (MathWorks, MA, USA). Subjective gating was set to exclude any cells/debris less than 3000 µm^2^ in cross-sectional area. For *BCL-3*-overexpression experiments, equal numbers of single SW620 cells stably transfected with pcDNA-BCL-3 WT vector or empty pcDNA vector as a negative control were seeded into Matrigel, and were cultured and analysed as described above.

### Tumoursphere-formation assay

For *BCL-3*-knockdown experiments, SW1463 cells transfected with *BCL-3* siRNA or negative-control siRNA were counted using Trypan blue (Invitrogen) and a Countess automated cell counter (Invitrogen) to exclude dead cells. A total of 2×10^4^ viable cells were re-suspended in tumoursphere medium consisting of DMEM:F12 medium (Gibco) supplemented with 20 ng/ml EGF (Sigma-Aldrich), 10 ng/ml FGF (R&D Systems), 2% B27 (Gibco), 400 µg/ml G418, 2 mM glutamine, 100 units/ml penicillin and 100 units/ml streptomycin (Invitrogen). Cells were seeded into non-adherent 6-well plates (Greiner Bio-One, Stonehouse, UK) and tumourspheres were cultured as described previously (Ricci-Vitiani et al., 2007) for 6 days before the contents of wells were centrifuged for 3 min at 3000 rpm (1400 ***g***). Tumourspheres were re-suspended in 500 µl of StemPro Accutase cell dissociation reagent (Gibco) and manually dissociated via pipetting. Cells were incubated for 30 min to achieve single-cell suspension and viable cells were counted using a Countess cell counter. For *BCL-3*-overexpression experiments, SW620 cells stably transfected with pcDNA-BCL-3 WT vector or empty pcDNA vector as a negative control were seeded, cultured and analysed as above.

### Statistical analysis

Statistical analysis was performed using a one-sample *t*-test or Student's *t*-test as stated. Significance was expressed as *P<0.05, **P<0.01 or ***P<0.001. Results are expressed as mean values with s.e.m.

## Supplementary Material

Supplementary information
