# Supplementary material for: BCL-3 promotes a cancer stem cell phenotype by enhancing β-catenin signalling in colorectal tumour cells
Source: Dis Model Mech. 2019 Mar 4;12(3):dmm037697. doi: 10.1242/dmm.037697 (PMC6451435; doi:10.1242/dmm.037697)
Supplement: Supplementary information [file dmm-12-037697-s1.pdf]

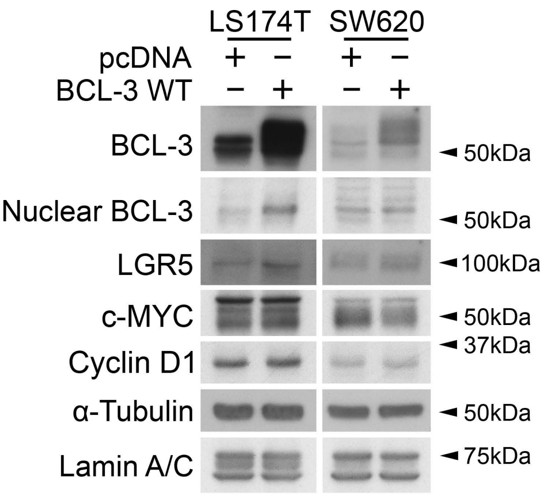

Figure S1. BCL-3 overexpression enhances LGR5 expression

LGR5 expression in LS174T and SW620 cells stably overexpressing BCL-3. LGR5, BCL-3, Cyclin D1 and c-MYC expression was analysed by western blot. α-Tubulin serves as a loading control. Nuclear BCL-3 expression was analysed in nuclear-enriched lysates with Lamin A/C serving as a loading control.

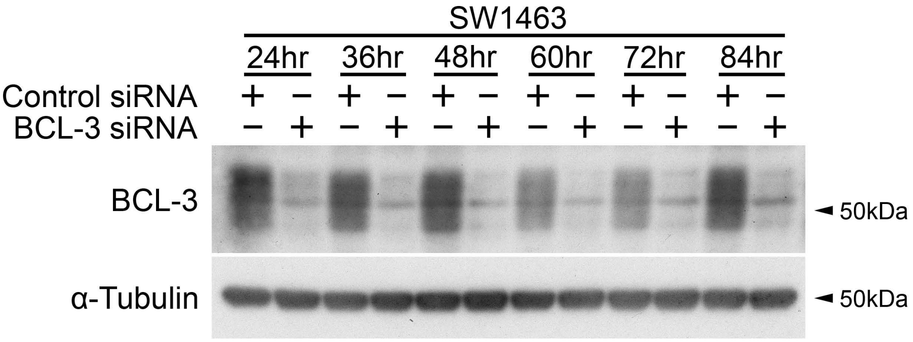

Figure S2. BCL-3 suppression is maintained at early stages of spheroid initiation

BCL-3 expression following 84 hours of BCL-3 knockdown in SW1463 cells grown as spheroids. α-Tubulin serves as loading control.
